# Survey to determine the farm‐level impact of Schmallenberg virus during the 2023–2024 UK lambing season

**DOI:** 10.1002/vetr.5595

**Published:** 2025-08-12

**Authors:** Sol Elliott, Rachel Clifton, Fiona Lovatt, Rachael Tarlinton

**Affiliations:** ^1^ School of Veterinary Medicine and Science University of Nottingham Loughborough UK; ^2^ Present address: Farm Gate Vets Lancaster UK

**Keywords:** bluetongue virus, ewes, lambs, Schmallenberg virus, surveys

## Abstract

**Background:**

This study aimed to assess the impacts of Schmallenberg virus (SBV) on the 2023/2024 UK lambing season.

**Methods:**

An online survey was distributed to UK sheep farmers between February and May 2024. Responses were compared across SBV‐confirmed, suspected and not suspected farms.

**Results:**

Higher impacts on flock welfare, financial performance and farmer emotional wellbeing were reported on SBV‐confirmed (*n* = 44) and SBV‐suspected (*n* = 84) farms compared to not suspected farms (*n* = 101), and higher dystocia and dystocia‐associated ewe deaths were reported on SBV‐confirmed farms. Lamb mortality was higher than in previous SBV outbreaks in the UK. An earlier mating season was identified as a risk factor for SBV.

**Limitations:**

Cross‐sectional surveys are prone to recall bias, and the data accuracy depends on the accuracy of farm records. Farmers who experienced negative impacts of SBV may have been more likely to complete the survey. There is also a risk of selection bias due to the study population being an opportunistic rather than a random sample of UK sheep farmers.

**Conclusion:**

This study confirms the significant impacts of SBV on the UK sheep industry, which are likely to be ongoing due to the cyclical re‐emergence pattern of the virus.

## INTRODUCTION

Schmallenberg virus (SBV) first emerged in Europe in 2011 and has since become endemic in parts of central Europe.^1^, ^2^ The virus is transmitted by *Culicoides* sp. midges. In adult cattle, it causes transient fever, diarrhoea and milk drop (though this is often not apparent), but clinical signs are not usually apparent in adult sheep. The major clinical and economic impact of the disease is due to cross‐placental infection of calves and lambs in mid‐gestation, causing neurological damage resulting in hydranencephaly and arthrogryposis.^3^

The UK experienced a large initial SBV outbreak in 2011/2012, with re‐emergence in 2016/2017 and 2023/2024.^4^, ^5^ As with other orthobunyaviruses, SBV‐induced deformities in ruminants in non‐endemic areas are normally seen in explosive 3–5‐year cycles.^6^ Seroconversion in the ruminant (cattle, sheep, deer) populations in Europe is usually high (>60%) in the year following a large outbreak, limiting circulation in the following years. As older seropositive animals are removed from the population, this population‐level immunity drops, resulting in a large population of naive animals again, and when weather conditions are suitable for large populations of the midge vector, a new large‐scale outbreak occurs.^1^, ^2^, ^7^, ^8^, ^9^, ^10^

Vaccination against the virus is effective at preventing infection and fetal deformities.^11^ However, the sporadic nature of outbreaks (both annually and at a national and flock level) means that use of the killed virus adjuvanted vaccines that were initially marketed in Europe was probably only cost effective for higher value dairy herds and pedigree beef herds and sheep flocks.^12^, ^13^ Vaccine uptake was never high,^5^ and there are no licensed vaccines currently available. Hence, management factors, such as moving the timing of mating so that the high‐risk periods for virus transmission and pregnancy do not coincide, are currently the only tools available for minimising the impact of the virus.^5^

SBV is not a notifiable disease in the UK or the European Union, and monitoring is not systematic; hence, it is thought that SBV is very much under‐reported.^14^ However, after an apparent prolonged absence, large‐scale circulation and subsequent fetal deformities were evident in the 2023/2024 lambing season in Europe.^15^, ^16^ Surveillance is largely by passive reporting and testing of abortion cases. An added complication in Northern Europe in 2023/2024 has been the emergence of bluetongue virus strain 3 (BTV‐3),^17^, ^18^ which can also cause cross‐placental infections with fetal deformities including hydraencephaly, though arthrogryposis is not usually seen with BTV. However, BTV does cause very high mortality in adult sheep^19^, ^20^, ^21^, ^22^ and is notifiable, with farms reporting cases subject to movement restrictions and subsequent economic losses due to inability to sell livestock. Both viruses are spread by the same *Culicoides* sp. biting midges, and it is expected that the eventual geographic range and spread of both viruses will be similar.^23^

Two papers have previously explored the impacts of SBV on UK sheep farmers during the 2011/2012 and 2016/2017 outbreaks.^5^, ^24^ These studies highlighted that the main impacts of the virus were increased lamb and ewe losses, with financial and emotional distress for affected farmers. These studies also showed that the impact of the virus was uneven, with a small number of flocks experiencing very high rates of lamb loss, and that flocks mating early were more likely to be affected. Early indicators in the UK were that SBV‐related losses in the 2023/2024 lambing season were very high (F. Lovatt, personal communication, 2024), and this study aimed to confirm this and identify the risk factors and economic impact associated with the virus as well as its geographical spread.

## MATERIALS AND METHODS

### Survey design

The survey was implemented as an online survey in Microsoft Forms (survey available in Supporting Information), with solicitation for responders promoted on social media by government services and farming organisations within the UK between February and May 2024. A link to the online questionnaire was also handed out by veterinary students while on Easter lambing placements. This recruitment strategy differed slightly from the 2011/2012 and 2016/2017 surveys,^5^, ^24^ as shown in Table 1. Questionnaire participation was voluntary.

**TABLE 1:** Comparison of recruitment strategies for the three studies of Schmallenberg virus outbreaks in the UK to date.

Harris et al (2011/2012)^24^	Stokes et al (2016/2017)^5^	Present study (2023/2024)
Online survey publicised by industry and government organisations. Invite letter sent to farmers who had submitted samples for SBV testing to AHVLA. Paper versions available for farmers without online access. Farmers able to complete survey at National Sheep Association biennial sheep event.	Online survey publicised by industry and government organisations. Invite letter sent to farmers who had submitted samples for SBV testing to APHA Link to the online questionnaire handed out by veterinary students while on Easter lambing placements.	Online survey publicised by industry and government organisations. Link to the online questionnaire handed out by veterinary students while on Easter lambing placements.

*Note*: AHVLA became APHA in 2014.

Abbreviations: AHVLA, Animal Health and Veterinary Laboratories Agency; APHA, Animal and Plant Health Agency.

To enable comparison with the 2011/2012 and 2016/2017 data, survey questions were designed to closely match those used in previous studies,^5^, ^24^ with some additional questions relating to BTV and vaccination intentions. In the current survey, farmers who did not suspect SBV in their flock were not asked about the number of ewes requiring assistance birthing deformed lambs or the nature of deformities that were seen in their lambs, as these questions received no responses from flocks where SBV was not suspected in the 2016/2017 survey. Farmers were able to add any additional comments they wished to share regarding the 2023/2024 lambing season as a free‐text entry. Ethical approval was granted by the University of Nottingham School of Veterinary Medicine and Science Committee for Animal Research and Ethics (CARE), number 4056 240124.

### Data analysis

All responses were downloaded from the MS Forms platform on 3 June 2024. Analyses were conducted in Microsoft Excel and R (v4.3.2).^25^ Initially, responses were checked for consistency, with any insufficiently completed responses removed from the working copy. These excluded responses included those from respondents who had not answered whether they believed their flock to have been infected by SBV in the 2023/2024 season, and those who had answered this question but not provided any other flock data.

Information about UK flock sizes was obtained from the 2019 Defra Agricultural Census^26^ to enable comparison of the survey respondents with the target population.

Each participating flock's SBV category was determined based on farmer responses as follows:

- SBV not suspected: farmer did not believe the flock to have been infected by SBV during 2023/2024 and had not sent samples for laboratory testing.
- SBV suspected: farmer believed the flock had been infected by SBV during 2023/2024, but this was not confirmed by laboratory testing.
- SBV confirmed: farmer believed the flock had been infected by SBV during 2023/2024, and this was confirmed by laboratory testing.

Any responses where SBV status could not be determined were removed from further analysis.

Outcome variables of interest were farm demography (farm and flock type), breeding season (month mating started, duration of breeding, duration of lambing), lambing productivity (scanning percentage, lambing percentage, barren rate), lamb and ewe mortality, number of ewes requiring assistance at parturition (farmer, vet or caesarean section), history of vaccination for SBV, farmer willingness to vaccinate, and farmers’ perceptions of the impact of SBV on sheep welfare, flock financial performance, farmers’ emotional wellbeing and their likelihood of farming sheep the following year. Not all farmers answered all questions; for each outcome variable, all participants who had answered that question were included in the analysis. The number of responses for each outcome is shown in Tables S1–S6.

Outcome variables were summarised across SBV categories using contingency tables for categorical variables and summary statistics (median, interquartile range, minimum and maximum) for continuous variables (Tables S1–S6). Distributions of continuous variables were visualised using histograms.

Analysis of variance and Tukey's honestly significant difference post hoc tests were used to compare differences across the SBV categories for normally distributed continuous data. Kruskal–Wallis tests and Dunn post hoc tests with Benjamini–Hochberg correction were used to compare differences across the SBV categories for continuous data with non‐normal distributions. Because the timing of mating was found to be strongly associated with SBV category, associations between other continuous outcome variables and SBV category were tested for confounding by mating start date using multivariable regression models (further details provided in Supporting Information and Table S7). Results from univariable analysis are presented unless multivariable analysis gave notably different results.

Chi‐squared tests and Fisher's exact tests were used to test for associations between SBV status and nominal categorical variables. An extended Cochran–Armitage test was used to check for associations between SBV status and ordered categorical variables.

### Mortality definitions

The same definitions of mortality and lambing percentage were applied as in the 2018 paper reporting data from the 2016/2017 lambing season:^5^

Lamb mortality (%) = 100*(lambs dead from any cause within 1 week/total lambs born)

Lambing mortality (%) = 100*(lambs dead from any cause within 1 week/number of non‐barren ewes)

Lambing percentage = 100*([Total number of lambs {dead+reared}]/[number of tupped ewes‐number of barren ewes])

Ewe mortality (%) = 100*(number of ewes that died during lambing/number of non‐barren ewes)

Excess lamb mortality was calculated as the difference between median lamb mortality on SBV‐confirmed and SBV‐not suspected farms. This was also calculated for the 2016/2017 and 2011/2012 outbreaks based on lamb mortality figures provided in previous publications.^5^, ^24^

### Malformation definitions

Malformations described by respondents were coded separately by S.E. and R.C. into the seven groups previously described^5^ and ‘Other’. S.E. and R.C. then compared codes and reached a consensus code for any descriptions that had been coded differently. Numbers of each malformation code were summarised for each SBV category (confirmed or suspected).

## RESULTS

### Farm demographics

In total, 247 respondents participated in the online survey, with 229 responses suitable for further analysis. Those not suitable for analysis either did not consent to the conditions of the study (*n* = 1), SBV category could not be determined (*n* = 14) or no flock data had been provided (*n* = 3). In total, 19.2% of respondents were from SBV‐confirmed farms, 36.7% from SBV‐suspected farms and 44.1% from SBV‐not‐suspected farms (*n* = 229). Table 2 provides a summary of the results of the current study alongside those from the 2011/2012 and 2016/2017 SBV outbreaks.

**TABLE 2:** Comparison of data from the three studies of Schmallenberg virus (SBV) outbreaks in the UK to date. Highlighted parameters are those that demonstrated a significant difference between SBV confirmed, suspected and not suspected farms.

Factor	Harris et al (2011/2012)^24^	Stokes et al (2016/2017)^5^	Present study (2023/2024)
Percentage of mated ewes that were empty	No difference in median numbers between SBV‐confirmed (4%), suspected (4.3%) and not suspected (3.3%) farms	No difference in median numbers between SBV‐confirmed (3.7%), suspected (4.3%) and not suspected (3.2%) farms	No difference between SBV‐confirmed (7.3%), suspected (4.8%) and not suspected (3.5%) farms
Mating season (start date and duration in days)	NA	Difference between mating start date groups between SBV categories	Difference between mating start date groups between SBV categories. Duration of mating shorter for not suspected than suspected farms. SBV‐confirmed farms not significantly different
Duration of lambing season (days)	No difference between SBV‐confirmed (49.5 days), suspected (48.5 days) and not suspected (44.5 days) farms	Difference between SBV‐confirmed (64.5 days), suspected (52.0 days) and not suspected (40.0 days) farms	Difference between SBV‐suspected (50.0 days) and not suspected (35.0 days) farms. SBV‐confirmed (35.0 days) not significantly different
Lambing percentage	No difference between SBV‐confirmed (169.1%), suspected (166.7%) and not suspected (164.2%) farms	No difference between SBV‐confirmed (174.3%), suspected (173.0%) and not suspected (166.7%) farms	No difference between SBV‐confirmed (146.6%), suspected (153.8%) and not suspected (168.6%) farms
Scanning percentage	NA	No difference between SBV‐confirmed (175.0%), suspected (172.5%) and not suspected (176.0%) farms	No difference between SBV‐confirmed (165.0%), suspected (170.0%) and not suspected (168.5%) farms
Lamb mortality	Higher mortality on SBV‐confirmed (10.4%) and suspected (7.0%) farms than on not suspected (5.3%) farms	Higher mortality on SBV‐confirmed (9.1%) and suspected (7.6%) farms than on not suspected (5.7%) farms	Higher mortality on SBV‐confirmed (19.6%) and suspected (15.4%) farms than on not suspected (7.6%) farms
Lambing mortality	Higher mortality on SBV‐confirmed (18.2%) and suspected (11.3%) farms than on not suspected (8.6%) farms	Higher mortality on SBV‐confirmed (15.2%) and suspected (12.7%) farms than on not suspected (8.4%) farms	Higher mortality on SBV‐confirmed (27.2%) and suspected (15.9%) farms than on not suspected (10.4%) farms
Percentage of farms with ≥1 breeding ewe that died during the lambing period	More SBV‐confirmed (66.7%) and SBV‐suspected (67.1%) farms with ewes dying than not suspected (54.5%) farms	No difference between SBV‐confirmed (71.4%), suspected (67.5%) and not suspected (59%) farms	No difference between SBV‐confirmed (47.6%), suspected (57.1%) and not suspected (58%) farms
Percentage of farms with ≥1 ewe that died giving birth to deformed lambs	More SBV‐confirmed (36.9%) and suspected 16.8%) farms with ≥1 ewe dying than not suspected (7.2%) farms	More SBV‐confirmed (30.9%) and suspected (28.4%) farms with ≥1 ewe dying than not suspected (5.6%) farms	More SBV‐confirmed (32.6%) and suspected (26.5%) farms with ≥1 ewe dying than not suspected (11.2%) farms
Percentage of farms with ≥1 ewe that gave birth to deformed lambs without assistance	NA	No difference between SBV‐confirmed (44.4%), suspected (52.9%) and not suspected (34.6%) farms	No difference between SBV‐confirmed (56.1%) and suspected (40.7%) farms
Percentage of farms with ≥1 ewe assisted by the farmer because of deformed lambs	NA	More SBV‐confirmed farms (80%) and suspected (78.2%) farms with ≥1 ewe assisted than not suspected (33.3%) farms	No difference between SBV‐confirmed (93%) and suspected (80.2%) farms
Percentage of farms with ≥1 ewe assisted by a vet because of deformed lambs	More SBV‐confirmed (35.8%) and suspected farms (19.5%) with ≥1 ewe assisted than not suspected (4.8%) farms	No difference between SBV‐confirmed (39.1%), suspected (33.3%) and not suspected (11.1%) farms	More SBV‐confirmed farms (53.3%) with ≥1 ewe assisted than suspected (20.7%) farms
Percentage of farms with ≥1 caesarean section because of deformed lambs	More SBV‐confirmed (12.3%) and suspected (11%) farms with ≥1 caesarean section than not suspected (1.6%) farms	More SBV‐confirmed (32.6%) and suspected (24.5%) farms with ≥1 caesarean section than not suspected (0%) farms	More SBV‐confirmed farms (32.6%) with ≥1 caesarean section than suspected (11.0%) farms
Farmer‐perceived impact of SBV on sheep welfare	Higher negative impact on SBV‐confirmed (36.8%) and suspected (17.8%) farms than on not suspected (0.5%) farms	Higher negative impact on SBV‐confirmed (79.3%) and suspected (59.3%) farms than on not suspected (10.5%) farms	Higher negative impact on SBV‐confirmed (100%) and suspected (92.9%) farms than on not suspected (6.1%) farms
Farmer‐perceived impact of SBV on financial performance	Higher negative impact on SBV‐confirmed (32.8%) and suspected (20.1%) farms than on not suspected (2.3%) farms	Higher negative impact on SBV‐confirmed (82.7%) and suspected (62.9%) farms than on not suspected (10.4%) farms	Higher negative impact on SBV‐confirmed (100%) and suspected (94%) farms than on not suspected (13.3%) farms
Farmer‐perceived impact of SBV on farmers' emotional wellbeing	Higher negative impact on SBV‐confirmed (49.3%) and suspected (25.6%) farms than on not suspected (6.5%) farms	Higher negative impact on SBV‐confirmed (70.7%) and suspected (61.7%) farms than on not suspected (33.3%) farms	Higher negative impact on SBV‐confirmed (100%) and suspected (89.3%) farms than on not suspected (41.8%) farms
Farmers less likely to farm sheep next year because of SBV	No difference between SBV‐confirmed (5.7%), suspected (5.9%) and not suspected (1.8%) farms	Higher percentage of farmers on SBV‐confirmed (10.2%) and suspected (3.7%) farms less likely to farm sheep next year than on not suspected (0%) farms	No difference between SBV‐confirmed (9.3%), suspected (8.3%) and not suspected (3.1%) farms

**FIGURE 1:**
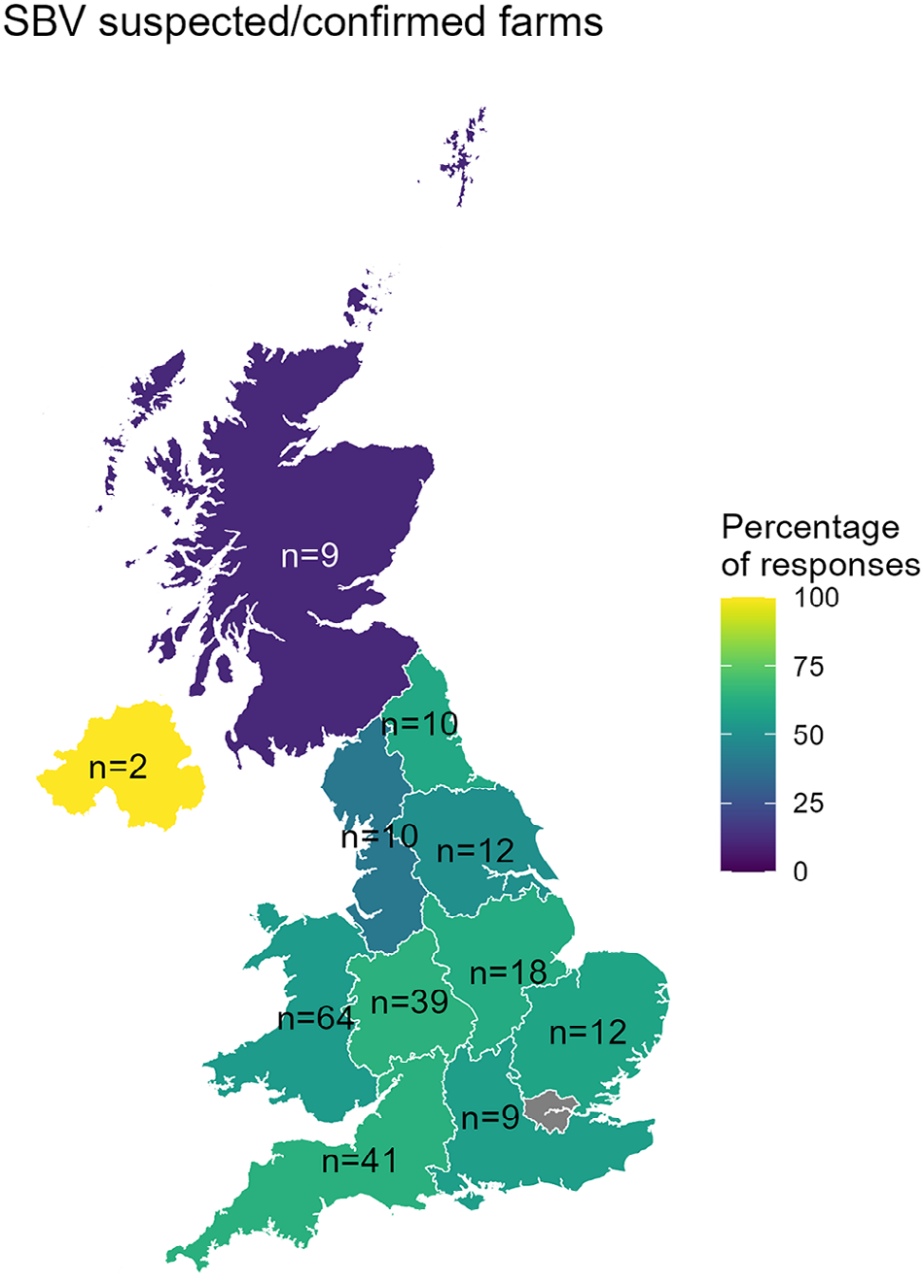
Percentage of responses by region that were from Schmallenberg virus (SBV)‐confirmed or SBV‐suspected farms. Total respondents included: SBV‐confirmed farms (*n* = 44), SBV‐suspected farms (*n* = 83), SBV‐not‐suspected farms (*n* = 99). One SBV‐suspected farm and two SBV‐not‐suspected farms did not provide a valid postcode and are therefore not shown on the map.

Most respondents were from the west of England and Wales (Figure 1), although cases were reported in Northern Ireland and the Scottish Lowlands. The average flock size for respondents was 216 breeding ewes, which is lower than the UK average of 350,^27^ and smaller flocks were slightly over‐represented in our data compared to all UK flocks (Figure S1). There was no difference in flock size between SBV categories (*p* = 0.95).

**FIGURE 2:**
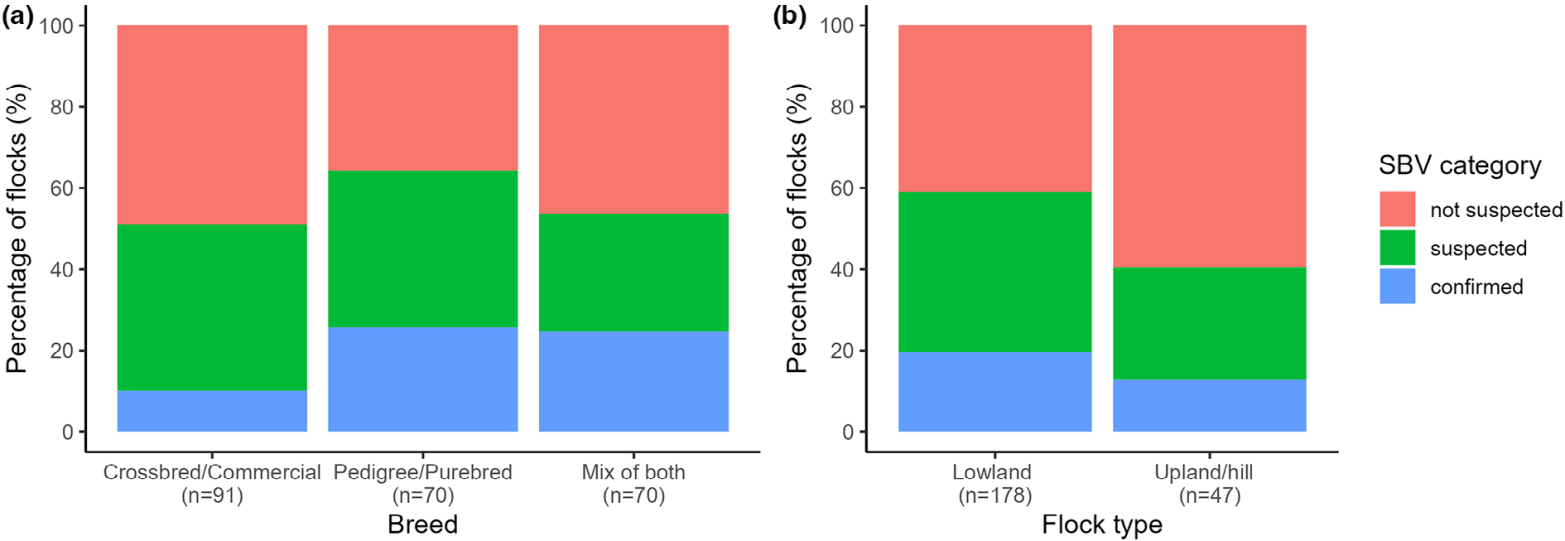
Percentage of crossbred/commercial, pedigree/purebred and mixed flocks (a), and lowland and upland flocks (b), in each Schmallenberg virus (SBV) category.

Overall, 39.0% of respondents defined their flock as crossbreeds/commercial animals, 30.3% were pedigree/purebred and 29.9% were a mix of both. Farms with pedigree/purebred sheep or a mix of both were more likely to be in the SBV‐confirmed category (*p* = 0.04), and there was a tendency for upland/hill farms (*n* = 47) to be in the SBV‐not‐suspected category (*p* = 0.07, Figure 2).

### Breeding seasons, scanning rates and lambing percentages

**FIGURE 3:**
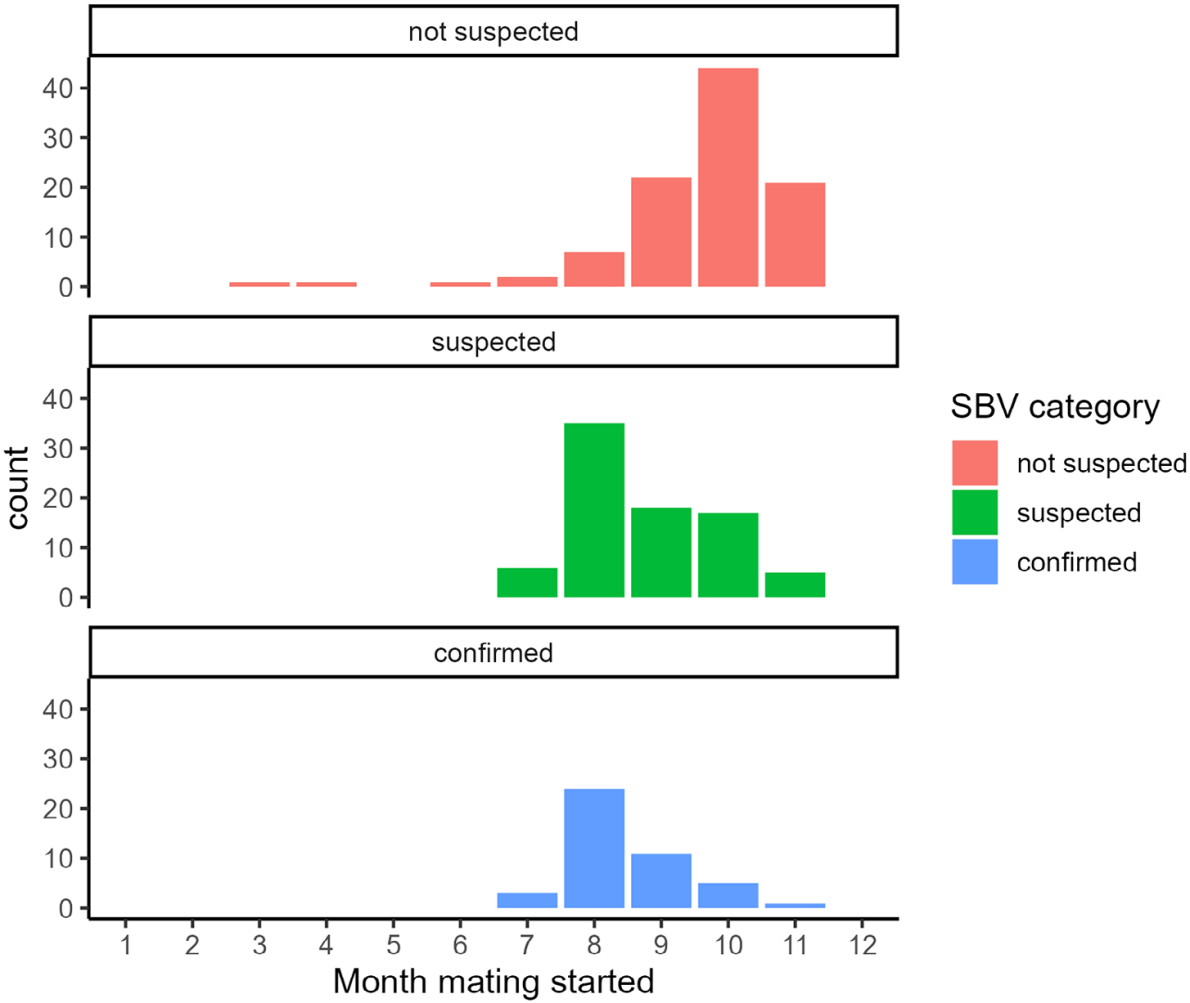
Distribution of the start of mating for Schmallenberg virus (SBV)‐not‐suspected, SBV‐suspected and SBV‐confirmed farms.

**FIGURE 4:**
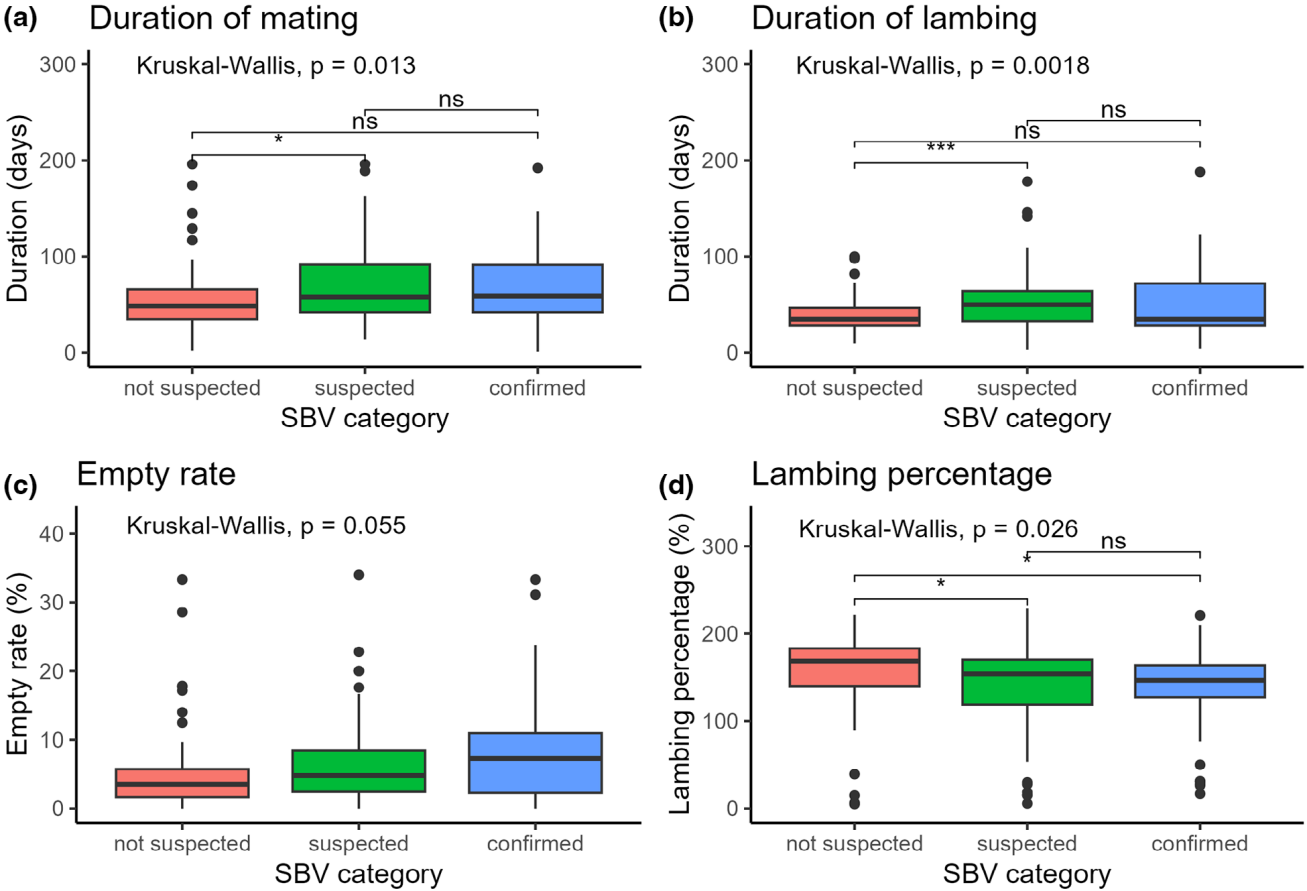
Duration of mating (a), duration of lambing (b), empty rate (percentage of total number of ewes) (c) and lambing percentage (number of lambs born per ewe) (d) in Schmallenberg virus (SBV)‐not‐suspected, suspected and confirmed flocks. ns = not significant, *p < 0.05, **p < 0.01, ***p < 0.001, ****p < 0.0001. The thick black bar shows the median, the filled box shows the interquartile range (IQR) and the whiskers extend to ±1.5× IQR. Outliers are shown as points above or below the whiskers.

SBV‐confirmed and SBV‐suspected farms started mating earlier (*p *< 0.001) than SBV‐not‐suspected farms (Figure 3); this was also observed in the 2016/2017 outbreak (Table 2). The duration of both mating and lambing was significantly longer on SBV‐suspected farms than on SBV‐not‐suspected farms (*p* = 0.018 and *p *< 0.001, respectively) (Figure 4a,b). The duration of mating and lambing also tended to be longer on SBV‐confirmed farms than on SBV‐not‐suspected farms, but this difference was not statistically significant.

There was a tendency for higher empty rates (Figure 4c) and lower scanning rates in SBV‐confirmed flocks compared to suspected and not suspected flocks, but this difference was not statistically significant (Tables S2 and S7). In univariable analysis, lambing percentages were lower on SBV‐suspected and confirmed farms than on SBV‐not‐suspected farms (*p* = 0.03, median 146.6%, 153.8% and 168.6%, respectively) (Figure 4d). However, this association was no longer significant once confounding by mating start date was accounted for (*p* = 0.052, Table S7).

### Lamb and lambing mortality

**FIGURE 5:**
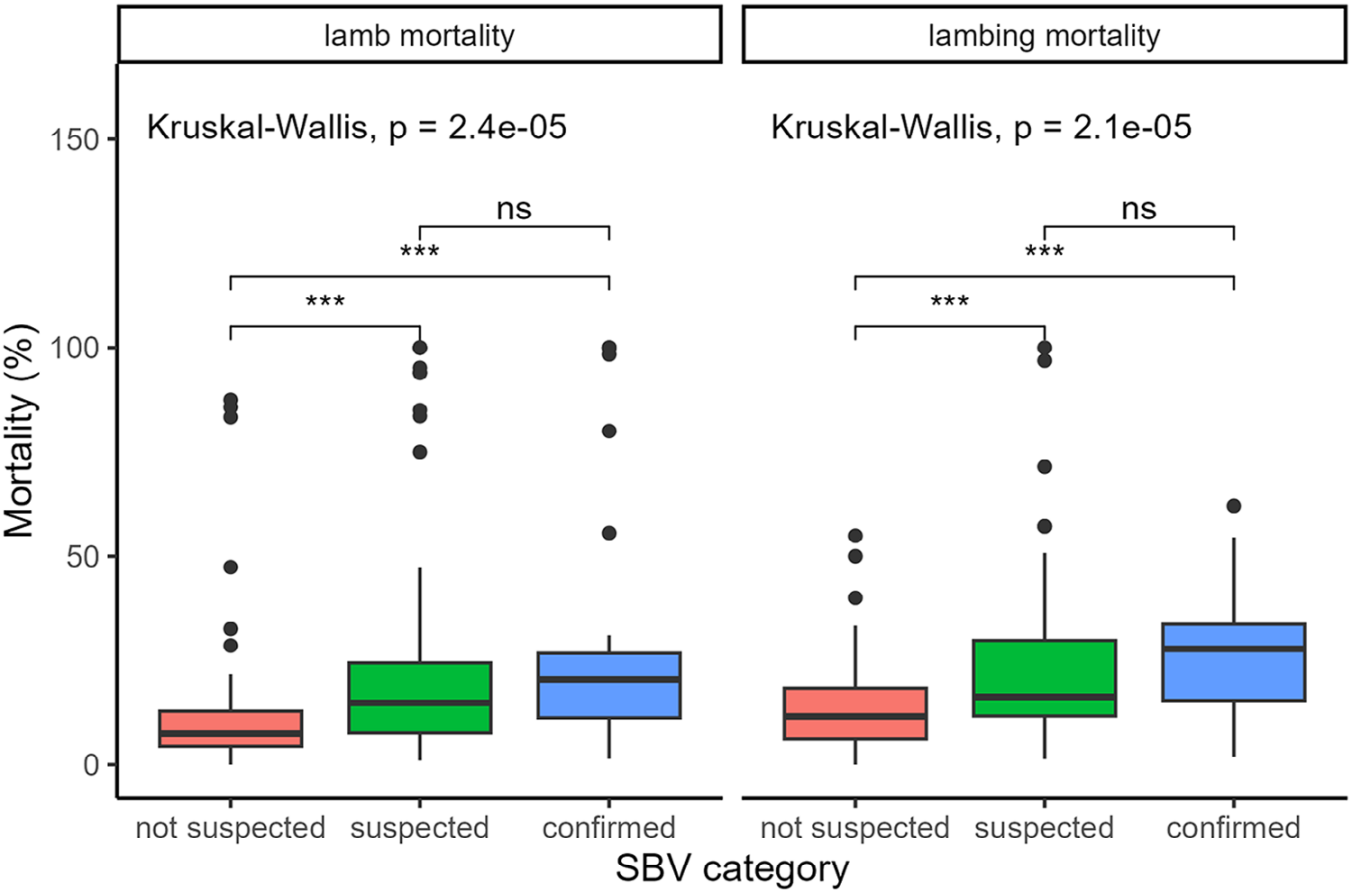
Lamb and lambing mortality for each Schmallenberg virus (SBV) category: lamb mortality = lamb deaths/number of lambs born, lambing mortality = lamb deaths/number of pregnant ewes. ns = not significant, *p < 0.05, **p < 0.01, ***p < 0.001, ****p < 0.0001. The thick black bar shows the median, the filled box shows the interquartile range (IQR) and the whiskers extend to ±1.5× IQR. Outliers are shown as points above or below whiskers.

There was higher lamb mortality (*p* < 0.001) on SBV‐confirmed and SBV‐suspected farms compared to SBV‐not‐suspected farms (median 19.6%, 15.4% and 7.6%, respectively) (Figure 5). This was also true for lambing mortality (median 27.2%, 15.9% and 10.4%, respectively). Both lamb and lambing mortality were higher across all three SBV categories than in the 2011/2012 and 2016/2017 SBV outbreaks (Table 2).^5^, ^24^

Particularly high lambing mortality (>40%) was observed more frequently on SBV‐confirmed farms (15.8%) and SBV‐suspected farms (11.7%) than on SBV‐not‐suspected farms (2.7%) (*p* = 0.03). This pattern was also observed for lamb mortality, but was not statistically significant (*p* = 0.09). Excess lamb mortality in SBV‐confirmed farms compared to SBV‐not‐suspected farms was 12%, which was higher than in previous outbreaks (3.4% and 5.1% for the 2016/2017 and 2011/2012 outbreaks, respectively).

### Abnormalities in lambs

At least one malformation was described on 84.1% (37/44) of SBV‐confirmed farms and 85.7% (72/84) of SBV‐suspected farms. The most common deformity reported by farmers was fused joints (SBV confirmed 28/44, 63.6%; SBV suspected 44/84, 52.3%), jaw deformities (SBV confirmed 17/44, 38.6%; SBV suspected 21/84, 25%) and twisted limbs (SBV confirmed 9/44, 20.5%; SBV suspected 16/84, 19.0%). Other common deformities on SBV‐suspected farms included bent necks (*n* = 14) and legs in abnormal places (*n* = 10).

### Ewe losses

Ewe mortality during the lambing period was not significantly different across the SBV categories (*p* = 0.50); however, more respondents from SBV‐confirmed and SBV‐suspected farms reported ewe deaths due to birthing a malformed lamb than respondents from SBV‐not‐suspected farms (32.6%, 26.5% and 11.2% of respondents, respectively, *p *< 0.01).

More respondents from SBV‐confirmed farms than SBV‐suspected farms had at least one ewe assisted by a vet because of a deformed lamb (53.5% vs. 20.7%) (*p *< 0.001). More respondents from SBV‐confirmed farms also reported at least one caesarean section due to birthing a deformed lamb compared to SBV‐suspected farms (32.6% vs. 11.0%, *p *< 0.01). Although slightly more respondents from SBV‐confirmed than SBV‐suspected farms reported having at least one ewe assisted by the farmer because of birthing a deformed lamb (93.0% vs. 80.2%), this was not a statistically significant difference (*p* = 0.07).

### Farmer‐perceived impact

**FIGURE 6:**
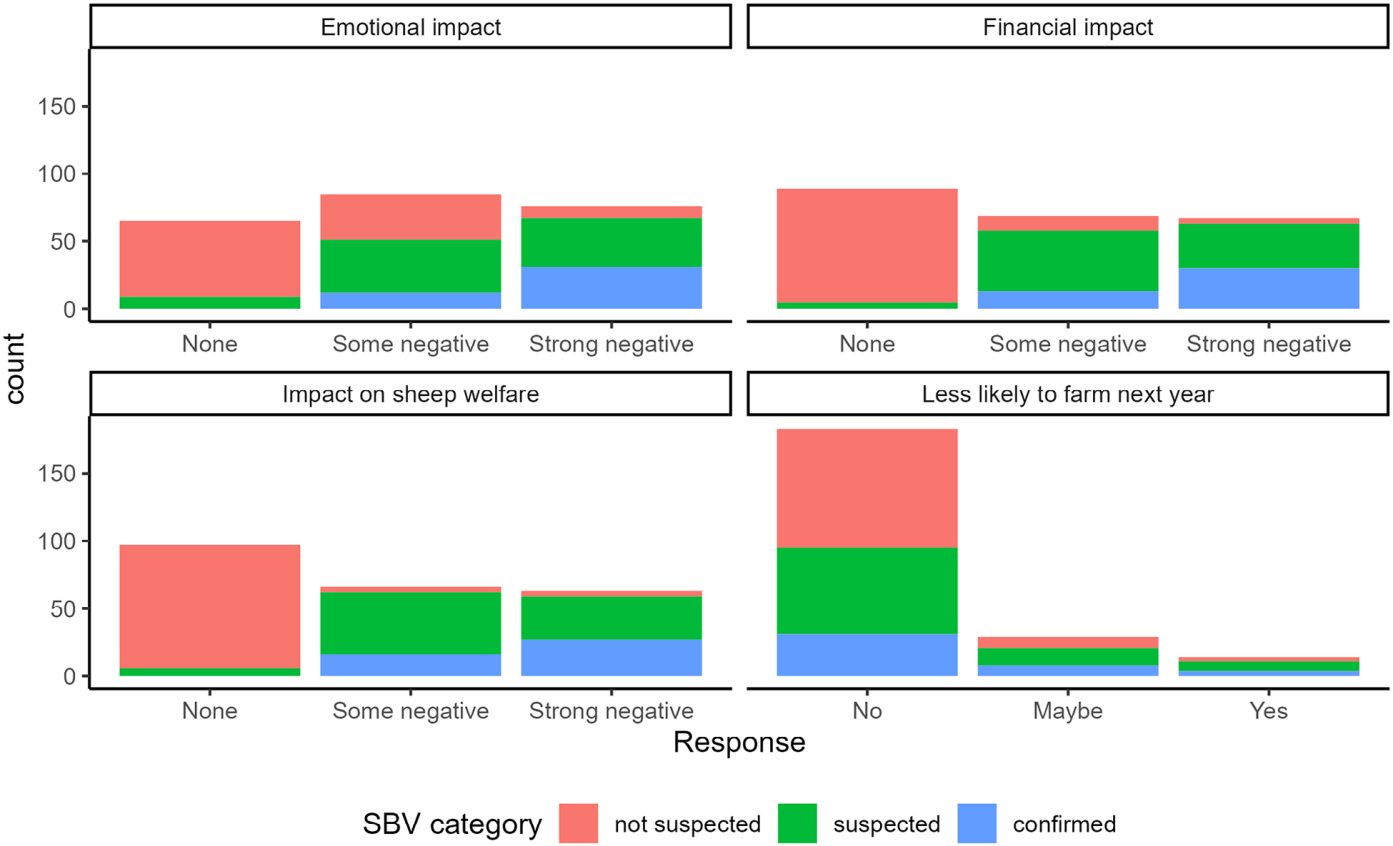
Farmer‐perceived emotional and financial impact of Schmallenberg virus (SBV) on SBV‐not‐suspected, SBV‐suspected and SBV‐confirmed farms.

There were significant differences in farmers perceptions of the impact of SBV on the welfare of the flock, financial performance of the flock and farmers' emotional wellbeing across SBV categories (all *p *< 0.001, Figure 6).

There were more respondents from SBV‐confirmed and SBV‐suspected farms who reported they were less likely to farm sheep the following year because of SBV, although this difference was not statistically significant (*p* = 0.08, 9.3% SBV confirmed, 7.1% SBV suspected and 1.0% SBV not suspected). Comments from respondents highlighting how difficult the 2023/2024 lambing season was for them include:

‘It's been utterly utterly soul destroying to the farm, to the sheep and to my partner's mental health’.

‘The economic cost is devastating’.

‘Despite no deformed lambs, the worry of the potential on us made for an emotionally worrying time until the last was done, it has made lambing far less enjoyable, and as hobby breeders of rare breeds, the worry of people not breeding in future is a concern’.

‘I never wish to have such a traumatic lambing again. So very depressing’.

‘This virus has had a huge financial impact on us and left us with a huge debt to the vet’.

‘After our dreadful lambing last year, largely as a result of SBV, we are seriously considering stopping lambing sheep in future’.

### SBV vaccination history and current demand

**FIGURE 7:**
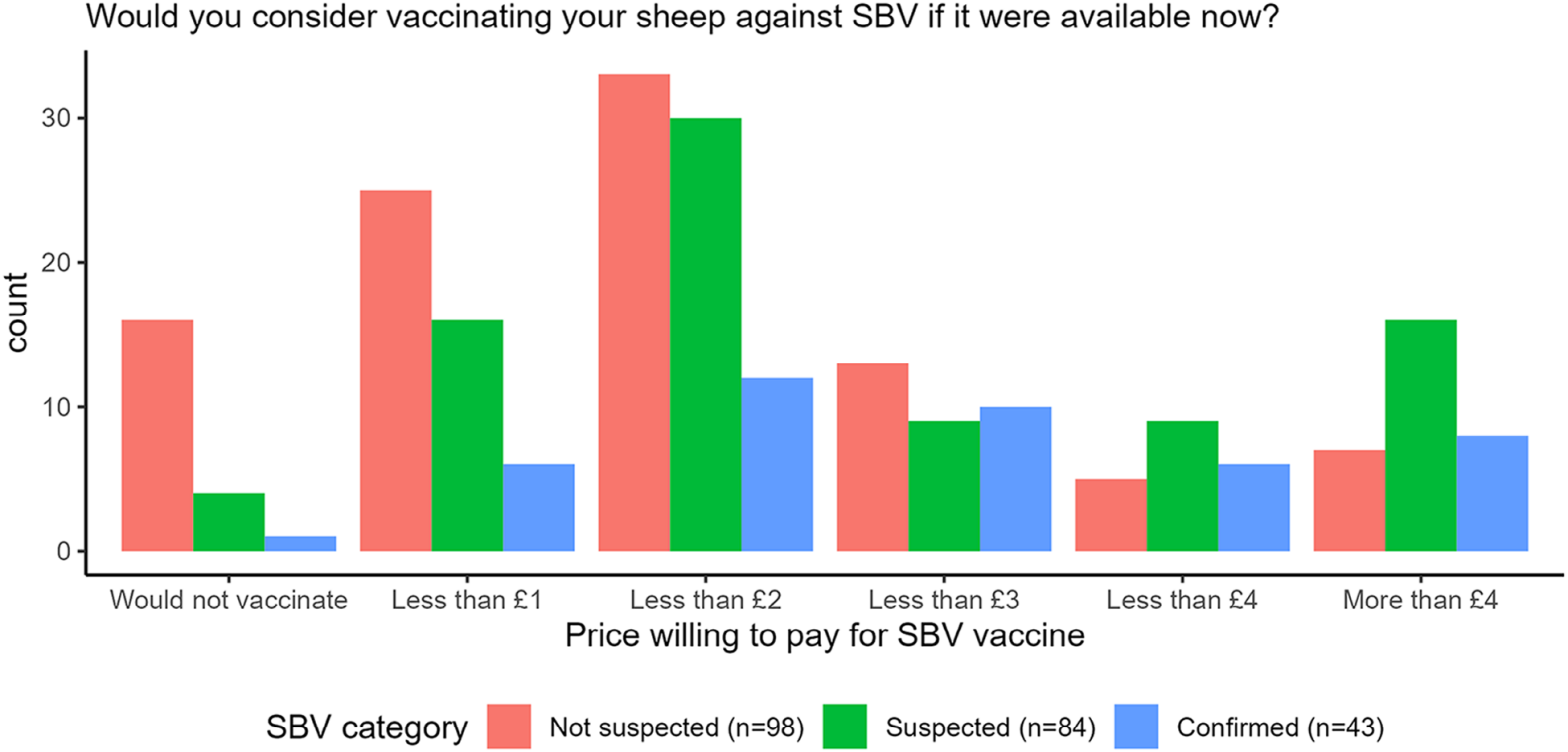
Vaccination intentions for Schmallenberg virus (SBV)‐not‐suspected, SBV‐suspected and SBV‐confirmed farms.

There was no difference in reported vaccination history between SBV categories (*p* = 0.85). There was a significant difference in the price respondents would be willing to pay to vaccinate against SBV between the SBV categories (*p* < 0.001, Figure 7). More respondents from SBV‐not‐suspected farms stated they would not vaccinate (16.3%) than respondents from SBV‐confirmed (2.3%) or SBV‐suspected farms (4.8%). The most common choice made by respondents, if they were to vaccinate, was to spend less than £2 a dose (Figure 7).

### Bluetongue

Only three farms reported suspicion of BTV‐3; however, after reviewing the locations of these farms and the known BTV‐3 cases in autumn 2023, it was deemed very unlikely that these were BTV‐3 cases. While indicative of farmer uncertainty and confusion about BTV and SBV, we have not analysed these further due to the uncertainty of the reports.

Of 211 respondents to the question regarding farmers' current willingness to vaccinate against BTV, 48 (22.7%) would not vaccinate, 44 (20.9%) would pay less than £1 to vaccinate each animal, 62 (29.4%) would pay less than £2, 20 (9.5%) would pay less than £3, 13 (6.2%) would pay less than £4 and 24 (11.4%) would pay over £4.

## DISCUSSION

SBV is now considered endemic in continental Europe, with variation in annual incidence and seroconversion.^1^, ^7^, ^23^, ^28^, ^29^, ^30^, ^31^ High levels of seroconversion follow large outbreaks, then annual circulation declines in subsequent years until conditions are right for another large‐scale outbreak.^32^ The last large outbreak of SBV in the UK was in 2016/2017, with sporadic cases and a small uptick in fetal deformities in 2021/2022.^33^ High levels of SBV were circulating in *Culicoides* midge pools in Germany in autumn 2023, and early indications from the 2023/2024 lambing season in the UK suggested that another large outbreak was underway.^4^, ^28^ SBV seropositive animals and positive SBV bulk milk samples were found in the UK in autumn 2023.^34^, ^35^ Between December 2023 and the end of March 2024, 233 lamb deformity cases sent to the APHA were positive for SBV, further supporting the timeline for virus circulation in the UK.^34^

This study compares data on the impacts of SBV on sheep farms from the 2011/2012 and 2016/2017 outbreaks, taking advantage of the use of survey data across the three periods to provide a fuller picture of the impact of the virus during large outbreak years (Table 2). Most of the parameters assessed were similarly affected during all three outbreak periods, though the scale of the outbreak and impact were different between the three outbreak cycles. This multicycle comparison of impacts gives confidence in predictions of the economic impacts of the virus for ongoing economic and epidemiological studies of SBV in ruminants in Europe.

Lamb and lambing mortality were higher in the current study than in previous outbreaks (Table 2). There were some minor methodological differences between these three studies, which theoretically could account for differences in the results. However, given the magnitude of the difference in lamb mortality between studies (median 19.6%, 9.1% and 10.4% for 2023/2024, 2026/17 and 2011/2012, respectively), this is unlikely to be due to methodological differences alone. As in the previous two outbreaks, the spread of impact across farms was very uneven, with a small number of farms experiencing extreme rates of lamb mortality (40–100%).

The 2023/2024 season was a very poor lambing season in the UK, with lamb mortality in SBV‐not‐suspected flocks higher than in previous years (Table 2). Wet weather was widely commented on in the free comments section of the survey. The UK meteorological office recorded the sixth wettest spring on record in 2024, with 302 mm total rainfall compared to 223 mm in 2017 and 231 mm in 2012.^36^, ^37^ However, excess lamb mortality in SBV‐confirmed versus SBV‐not‐suspected farms was also greater in 2023/2024 than in 2016/2017 and 2011/2012 (12%, 3.4% and 5.1%, respectively), suggesting that weather was not solely responsible for the higher lamb mortality in 2023/2024. More SBV‐confirmed and SBV‐suspected flocks mated (and therefore lambed) early (Figure 3); thus, it is possible that bad weather impacted these flocks more severely than flocks that lambed later. However, there was still a significant difference in lamb mortality between SBV categories even when accounting for the timing of mating/lambing.

Impacts on conception rates have been documented in dairy cattle^38^, ^39^ affected by SBV, and this is consistent with viral interference with maternal recognition of pregnancy (which in ruminants is mediated by interferon [IFN] tau), as documented for other viral diseases such as bovine viral diarrhoea virus (BVDV) and BTV.^40^ The extended periods of natural mating (giving multiple chances for conception) have made this harder to document in sheep. Indeed, in this and the previous surveys, differences in scanning percentages (ultrasound‐assessed pregnancy rates and litter size assessment in mid gestation) were not apparent between SBV‐confirmed, SBV‐suspected and SBV‐not‐suspected flocks. Differences in male fertility parameters could contribute to effects on conception rates, and SBV is excreted intermittently in bull semen,^40^, ^41^ though this has not been reported in the limited studies on sheep to date.^42^, ^43^

One of the factors in SBV epidemiology that is consistent across both the 2016/2017 and 2023/2024 surveys (not assessed in the 2011/2012 survey) is that the timing of mating is a critical risk factor in SBV outbreaks, with early‐mating flocks more likely to be affected. In this study, SBV‐confirmed and SBV‐suspected farms typically started mating in July/August (61.4% and 51.2%, respectively), whereas the majority of SBV‐not‐suspected farms (89.8%) reported mating in September–November. This supports the idea that ewes that were mated earlier would have been within the vulnerable period of gestation (approximately days 28–56 of pregnancy) in August, September and October. Midge numbers in the UK usually peak in August/September,^44^ and previous studies have demonstrated SBV viraemia and/or seroconversion in September/October.^45^ No breed differences in SBV risk have been seen, but in both the 2023/2024 and 2016/2017 surveys, hill flocks were less likely to be affected. However, this is difficult to untangle from the timing of mating and lambing, as hill flocks in the UK tend to be mated later than lowland flocks.

In all three outbreaks, farms reported significant financial and animal and human welfare impacts of SBV. This led to higher numbers of farmers considering leaving sheep farming altogether. Defra's census in 2023 showed a 5.1% reduction in the UK sheep flock, which is expected to continue to decline with the impacts of SBV on sheep farmers and their livestock.^46^ These reported impacts were greater in SBV‐confirmed and SBV‐suspected flocks than SBV‐not‐suspected flocks, likely leading to the greater willingness to vaccinate and to pay more for vaccination. Willingness to vaccinate and willingness to pay higher costs were stronger in 2023/2024 than in 2016/2017, reflecting the larger outbreak and impacts in 2023/2024.

A limitation of this study is that we cannot be sure how representative our sample of farmers was of the wider UK population. Our sample had a lower average flock size than the target population, which could reduce the generalisability of the results. As with all surveys, ‘volunteer bias’ may have occurred, leading to over‐representation of respondents with negative experiences of SBV. However, there was good representation from farmers who did not suspect SBV in their flocks (44.1% of respondents), and the distribution of our respondents is very similar to the national flock numbers. Because the survey was distributed primarily by social media, there may have been under‐representation from farmers who do not access these channels. Finally, surveys also have the potential for recall bias, leading to inaccuracies in the data obtained. We mitigated for this as far as possible by running the survey during the 2023/2024 lambing season when events would be fresh in farmers’ minds.

Overall, this study confirms the significant impacts of SBV on the viability of sheep farming in the UK. Lamb mortality due to SBV in 2023/2024 was very high, with an excess mortality of 12% in SBV‐confirmed flocks. The sporadic nature of these large outbreaks and the unpredictability of individual farm impacts make it difficult to plan a long‐term disease reduction strategy. The only effective preventive measure is vaccination, but due to poor uptake, these are no longer available in Europe.^47^ This study confirms that mating timing is critical for SBV impact, with later mating flocks less affected. Shifting of breeding to later in the year could mitigate the impacts of SBV, but this would have economic impacts as earlier lambs receive a price premium compared to those born later in the season.^48^

For the 2024/2025 lambing season, we would expect that SBV impacts would be lower due to high seroconversion of adult ewes in 2023/2024. Confirmation of this with serological studies would be useful for the certainty of prediction.

To complicate matters, BTV‐3 circulated through much of the east of England in autumn 2024 and is expected to re‐emerge in 2025, either from overwintering or being blown over with midges from the continent.^49^, ^50^ There is an overlap in effects on fetuses^51^ in mid‐gestation between SBV and BTV and the endemic BVDV/Border disease virus, with all three viruses causing hydranencephaly, presenting as blind or ‘dummy’ calves and lambs unable to suckle or stand. We would expect to see some BTV‐3‐affected lambs in 2024/2025, and these will be difficult to distinguish from SBV‐affected lambs without specific testing.

## AUTHOR CONTRIBUTIONS

Rachael Tarlinton and Fiona Lovatt conceived and designed the project and acquired the data. Sol Elliott and Rachel Clifton analysed the data.

## CONFLICT OF INTEREST STATEMENT

The authors declare no conflicts of interest.

## FUNDING INFORMATION

The authors received no specific information for this work.

## ETHICS STATEMENT

Ethical approval for this study was granted by the University of Nottingham School of Veterinary Medicine and Science Committee for Animal Research and Ethics (CARE), number 4056 240124.

## Supporting information



Supporting Information

Supporting Information

## Data Availability

The data that support the findings of this study are available on request from the corresponding author. The data are not publicly available due to privacy or ethical restrictions.

## References

[1] Kęsik‐Maliszewska J , Collins ÁB , Rola J , Blanco‐Penedo I , Larska M . Schmallenberg virus in Poland: endemic or re‐emerging? A six‐year serosurvey. Transbound Emerg Dis. 2021;68:2188–2198. 33012078 10.1111/tbed.13870

[2] Wernike K , Fischer L , Twietmeyer S , Beer M . Extensive Schmallenberg virus circulation in Germany, 2023. Vet Res. 2024;55:134. 39375811 10.1186/s13567-024-01389-5 PMC11460034

[3] Tarlinton R , Daly J , Dunham S , Kydd J . The challenge of Schmallenberg virus emergence in Europe. Vet J. 2012;194:10–18. 23026716 10.1016/j.tvjl.2012.08.017

[4] Tarlinton R , Clifton R , Lovatt F . Schmallenberg virus: lambing season survey. Vet Rec. 2024;194:156–157. 38362982 10.1002/vetr.3980

[5] Stokes JE , Tarlinton RE , Lovatt F , Baylis M , Carson A , Duncan JS . Survey to determine the farm‐level impact of Schmallenberg virus during the 2016–2017 United Kingdom lambing season. Vet Rec. 2018;183:690. 30257875 10.1136/vr.104866 PMC6312887

[6] APHA and SRUC . GB cattle quarterly report. 2021. https://assets.publishing.service.gov.uk/media/60c0b07fe90e07439684bda1/pub‐survrep‐c0121.pdf

[7] Wernike K , Fischer L , Holsteg M , Aebischer A , Petrov A , Marquart K , et al. Serological screening in wild ruminants in Germany, 2021/2022: no evidence of SARS‐CoV‐2, bluetongue virus or pestivirus spread but high seroprevalences against Schmallenberg virus. Transbound Emerg Dis. 2022;69:e3289–e3296. 35585653 10.1111/tbed.14600 PMC9348064

[8] Collins ÁB , Barrett DJ , Doherty ML , McDonnell M , Mee JF . Significant re‐emergence and recirculation of Schmallenberg virus in previously exposed dairy herds in Ireland in 2016. Transbound Emerg Dis. 2017;64:1359–1363. 28762657 10.1111/tbed.12685

[9] Stokes JE , Baylis M , Duncan JS . A freedom from disease study: Schmallenberg virus in the south of England in 2015. Vet Rec. 2016;179:435. 27729590 10.1136/vr.103903 PMC5136694

[10] King B , O'Shea Brown T , Tarlinton R , Daly JM . Seroprevalence of Schmallenberg virus in the United Kingdom and the Republic of Ireland: 2011–2013. Vet Microbiol. 2015;180:36–40. 26255555 10.1016/j.vetmic.2015.07.025

[11] Hechinger S , Wernike K , Beer M . Single immunization with an inactivated vaccine protects sheep from Schmallenberg virus infection. Vet Res. 2014;45:79. 25087007 10.1186/s13567-014-0079-6 PMC4237939

[12] Stavrou A , Daly JM , Maddison B , Gough K , Tarlinton R . How is Europe positioned for a re‐emergence of Schmallenberg virus? Vet J. 2017;230:45–51. 28668462 10.1016/j.tvjl.2017.04.009

[13] Waret‐Szkuta A , Alarcon P , Hasler B , Rushton J , Corbière F , Raboisson D . Economic assessment of an emerging disease: the case of Schmallenberg virus in France. Rev Sci Tech. 2017;36:265–277. 28926010 10.20506/rst.36.1.2627

[14] DEFRA and APHA . Schmallenberg virus. 2025. https://www.gov.uk/government/publications/schmallenberg‐virus/schmallenberg‐virus

[15] Schmallenberg: what you need to know. NFU Online; 2024. https://www.nfuonline.com/updates‐and‐information/schmallenberg‐what‐you‐need‐to‐know/#:~:text=Cases%20of%20Schmallenberg%20rose%20across,to%20be%20reported%20to%20Defra

[16] Surveillance Intelligence Unit . VIDA annual report 2022. 2023. https://public.tableau.com/app/profile/siu.apha/viz/VIDAAnnualReport2022/VIDAAnnualReport2022

[17] DEFRA and APHA . Bluetongue: news, information and guidance for livestock keepers. 2024. https://www.gov.uk/government/collections/bluetongue‐information‐and‐guidance‐for‐livestock‐keepers#:~:text=Between%20November%202023%20and%20March,in%20Kent%2C%20Norfolk%20and%20Suffolk

[18] DEFRA and APHA . Bluetongue: how to spot and report it. 2024. https://www.gov.uk/guidance/bluetongue

[19] Fairbanks EL , Daly JM , Tildesley MJ . Modelling the influence of climate and vector control interventions on arbovirus transmission. Viruses. 2024;16:1221. 39205195 10.3390/v16081221 PMC11359451

[20] van den Brink KMJA , Santman‐Berends IMGA , Harkema L , Scherpenzeel CGM , Dijkstra E , Bisschop PIH , et al. Bluetongue virus serotype 3 in ruminants in the Netherlands: clinical signs, seroprevalence and pathological findings. Vet Rec. 2024;195:e4533. 39148262 10.1002/vetr.4533

[21] Möhlmann TWR , Keeling MJ , Wennergren U , Favia G , Santman‐Berends I , Takken W , et al. Biting midge dynamics and bluetongue transmission: a multiscale model linking catch data with climate and disease outbreaks. Sci Rep. 2021;11:1892. 33479304 10.1038/s41598-021-81096-9 PMC7820592

[22] Santman‐Berends IMGA , van den Brink KMJA , Dijkstra E , van Schaik G , Spierenburg MAH , van den Brom R . The impact of the bluetongue serotype 3 outbreak on sheep and goat mortality in the Netherlands in 2023. Prev Vet Med. 2024;231:106289. 39126984 10.1016/j.prevetmed.2024.106289

[23] Tildesley MJ , Brand S , Brooks Pollock E , Bradbury NV , Werkman M , Keeling MJ . The role of movement restrictions in limiting the economic impact of livestock infections. Nat Sustain. 2019;2:834–840. 31535037 10.1038/s41893-019-0356-5 PMC6751075

[24] Harris KA , Eglin RD , Hayward S , Milnes A , Davies I , Cook AJC , et al. Impact of Schmallenberg virus on British sheep farms during the 2011/2012 lambing season. Vet Rec. 2014;175:172. 24795165 10.1136/vr.102295 PMC4145415

[25] R Core Team . R: A language and environment for statistical computing. Vienna, Austria: R Foundation for Statistical Computing; 2021.

[26] DEFRA . Farming statistics, final crop areas, yields, livestock populations and agricultural workforce of June 2019 ‐ United Kingdom. 2019. https://assets.publishing.service.gov.uk/media/5e4560dde5274a6d3205e826/structure‐jun2019final‐uk‐22jan20‐rev_v2.pdf

[27] AHDB . The breeding structure of the British sheep industry 2021. 2021. https://projectblue.blob.core.windows.net/media/Default/Beef%20&%20Lamb/SheepBreedSurvey4295_130821_WEB.pdf

[28] Voigt A , Kampen H , Heuser E , Zeiske S , Hoffmann B , Höper D , et al. Bluetongue virus serotype 3 and Schmallenberg virus in *C* *ulicoides* biting midges, Western Germany, 2023. Emerg Infect Dis. 2024;30:1438–1441. 38916645 10.3201/eid3007.240275 PMC11210651

[29] Milićević V , Sapundžić ZZ , Glišić D , Kureljušić B , Vasković N , Đorđević M , et al. Cross‐sectional serosurvey of selected infectious diseases in wild ruminants in Serbia. Res Vet Sci. 2024;170:105183. 38359648 10.1016/j.rvsc.2024.105183

[30] Bayrou C , Lesenfants C , Paternostre J , Volpe R , Moula N , Coupeau D , et al. Schmallenberg virus, cyclical reemergence in the core region: A seroepidemiologic study in wild cervids, Belgium, 2012–2017. Transbound Emerg Dis. 2022;69:1625–1633. 33949132 10.1111/tbed.14136

[31] Jiménez‐Martín D , Cano‐Terriza D , Díaz‐Cao JM , Pujols J , Fernández‐Morente M , García‐Bocanegra I . Epidemiological surveillance of Schmallenberg virus in small ruminants in southern Spain. Transbound Emerg Dis. 2021;68:2219–2228. 33034150 10.1111/tbed.13874

[32] Agerholm JS , Wernike K . Occurrence of malformed calves in April–May 2021 indicates an unnoticed 2020 emergence of Schmallenberg virus in Denmark. Transbound Emerg Dis. 2022;69:3128–3132. 34850578 10.1111/tbed.14405

[33] SRUC Veterinary Services . Schmallenberg virus transmission confirmed in north‐east Scotland. Vet Rec. 2022;190:232–234. 35303355 10.1002/vetr.1600

[34] APHA. Disease surveillance in England and Wales, April 2024. Vet Rec. 2024;194:339–342. 38700168 10.1002/vetr.4228

[35] APHA . GB cattle quarterly report, volume 28: quarter 1 (January to March) 2024. 2024. https://assets.publishing.service.gov.uk/media/60c0b07fe90e07439684bda1/pub‐survrep‐c0121.pdf

[36] Met Office . Warm May and spring for the UK. 2024. https://www.metoffice.gov.uk/about‐us/news‐and‐media/media‐centre/weather‐and‐climate‐news/2024/warm‐may‐and‐spring‐for‐the‐uk#:~:text=Spring%202024's%20average%20mean%20temperature,on%20record%20by%20mean%20temperature

[37] Met Office National Climate Information Centre . Areal values from HadUK‐Grid 1km gridded climate data from land surface network. 2025.

[38] Lechner I , Wüthrich M , Meylan M , van den Borne BHP , Schüpbach‐Regula G . Association of clinical signs after acute Schmallenberg virus infection with milk production and fertility in Swiss dairy cows. Prev Vet Med. 2017;146:121–129. 28992916 10.1016/j.prevetmed.2017.07.020

[39] Veldhuis AMB , Santman‐Berends IMGA , Gethmann JM , Mars MH , van Wuyckhuise L , Vellema P , et al. Schmallenberg virus epidemic: Impact on milk production, reproductive performance and mortality in dairy cattle in the Netherlands and Kleve district, Germany. Prev Vet Med. 2014;116:412–422. 24880623 10.1016/j.prevetmed.2014.04.015

[40] Wathes DC , Oguejiofor CF , Thomas C , Thomas C . Importance of viral disease in dairy cow fertility. Engineering. 2020;6:26–33. 32288965 10.1016/j.eng.2019.07.020 PMC7104734

[41] Schulz C , van der Poel WHM , Ponsart C , Cay AB , Steinbach F , Zientara S , et al. European interlaboratory comparison of Schmallenberg virus (SBV) real‐time RT‐PCR detection in experimental and field samples: the method of extraction is critical for SBV RNA detection in semen. J Vet Diagn Invest. 2015;27:422–430. 26185122 10.1177/1040638715593798

[42] Laloy E , Riou M , Barc C , Belbis G , Bréard E , Breton S , et al. Schmallenberg virus: experimental infection in goats and bucks. BMC Vet Res. 2015;11:221. 26297244 10.1186/s12917-015-0516-4 PMC4546222

[43] Curwen A , Jones S , Stayley C , Eden L , McKay H , Davies P , et al. Failure to detect Schmallenberg virus RNA in ram semen in the UK (2016–2018). Vet Rec Open. 2022;9:e39. 35770041 10.1002/vro2.39 PMC9208715

[44] White SM , Sanders CJ , Shortall CR , Purse BV . Mechanistic model for predicting the seasonal abundance of *Culicoides* biting midges and the impacts of insecticide control. Parasit Vectors. 2017;10:162. 28347327 10.1186/s13071-017-2097-5 PMC5369195

[45] Jones S , Eden L , McKay H , Bollard N , Dunham S , Davies P , et al. Schmallenberg virus neutralising antibody responses in sheep. BMC Vet Res. 2019;15:426. 31779623 10.1186/s12917-019-2139-7 PMC6883675

[46] AHDB . Defra's December survey reveals a drop in UK sheep and cattle populations. 2024. https://ahdb.org.uk/news/defra-december-survey-reveals-a-drop-in-uk-sheep-and-cattle-populations#:~:text=According%20to%20Defra's%20latest%20census,year%2C%20to%2021.2%20million%20head

[47] Wernike K , Beer M . More than a decade of research on Schmallenberg virus—knowns and unknowns. Adv Virus Res. 2024;120:77–98. 39455169 10.1016/bs.aivir.2024.09.003

[48] DAERA . Advancing the breeding season and increasing production in early lambing flocks. 2020. https://www.daera-ni.gov.uk/news/advancing‐breeding‐season‐and‐increasing‐production‐early‐lambing‐flocks

[49] DEFRA . Bluetongue virus in Europe, updated outbreak assessment #11. 2024. https://assets.publishing.service.gov.uk/media/66e151e0865c0eef0bc42dfd/Updated_Outbreak_Assessment__11_Bluetongue_Virus_in_Europe.pdf

[50] AHDB . Bluetongue virus latest news. 2024. https://ahdb.org.uk/bluetongue

[51] Golender N , Bumbarov V , Kovtunenko A , David D , Guini‐Rubinstein M , Sol A , et al. Identification and genetic characterization of viral pathogens in ruminant gestation abnormalities, Israel, 2015–2019. Viruses. 2021;13:2136. 34834943 10.3390/v13112136 PMC8619439

