# Supplementary material for: Survey to determine the farm‐level impact of Schmallenberg virus during the 2023–2024 UK lambing season
Source: Vet Rec. 2025 Aug 12;197(4):e5595. doi: 10.1002/vetr.5595 (PMC12355902; doi:10.1002/vetr.5595)
Supplement: Supplementary file 1 — Supporting Information [file VETR-197-e5595-s001.pdf]

1. I am over 16 years of age and consent to the conditions of participation in this study *

- ◯ yes
- ◯ no

Flock Details

2. What are the first two letters of your postcode? *

3. What type of flock do you own?

- ◯ Lowland
- ◯ Upland/hill

4. Is your flock

- ◯ Pedigree/Purebred
- ◯ Crossbred/Commercial
- ◯ Mix of both

5. What breed are your ewes?

Breeding, Fertility and Lambing Details 2023/24 breeding season

6. How many breeding ewes did you put to the ram ? *

The value must be a number

7. What date did the rams go in with the ewes?

8. What date were the rams taken out?

9. Were the ewes scanned during pregnancy?

- yes
- no

10. If the ewes were scanned in pregnancy please fill in your date of scanning

11. What was your scanning percentage this lambing season ?

The value must be a number

12. What was your scanning percentage last lambing season (2022/23)?

The value must be a number

13. How many barren ewes did you have at scanning this season ?

The value must be a number

14. What date did lambing start for this season?

15. What date did lambing end, or is lambing expected to end, this season ?

Total Lamb and Ewe Losses 2023/24 Season

16. Please enter the total number of lambs that:
    were aborted or were still born

    The value must be a number

17. Please enter the total number of lambs that:
    died within 1 week of birth

    The value must be a number

18. Please enter the total number of lambs that:
    were reared for more than 1 week

    The value must be a number

19. Please give the total number of breeding ewes that:
    died during the lambing period

    The value must be a number

20. Total number of breeding ewes that:
    died during lambing because of difficulties giving birth to deformed lambs

    The value must be a number

Lamb Losses due to Schmallenberg and Bluetongue 2023/24

21. Do you believe your flock was infected by Schmallenberg or Bluetongue this year (2023/24)?

- ◯ Yes Schmallenberg only
- ◯ Yes Bluetongue only
- ◯ Yes both
- ◯ No

22. Was this confirmed by laboratory testing?

- ◯ Yes for Schmallenberg only
- ◯ Yes for Bluetongue only
- ◯ Yes for Both
- ◯ No for Both

23. Please enter the number of Schmallenberg affected lambs (suspected or confirmed) that:

were aborted or were still born

[ ]

The value must be a number

24. Please enter the number of Schmallenberg affected lambs (suspected or confirmed) that:

died within 1 week of birth

[ ]

The value must be a number

25. Please enter the number of Schmallenberg affected lambs (suspected or confirmed) that:

were reared for for more than 1 week

[ ]

The value must be a number

26. Please enter the number of Bluetongue affected lambs (suspected or confirmed) that:

were aborted or stillborn

[ ]

The value must be a number

27. Please enter the number of Bluetongue affected lambs (suspected or confirmed) that:
died within 1 week of birth

The value must be a number

28. Please enter the number of Bluetongue affected lambs (suspected or confirmed) that:
were reared for more than 1 week

The value must be a number

29. If you have had malformed lambs please describe the types of problems they had

30. For ewes that produced one or more deformed lambs please give us the number that:
lambed on their own

The value must be a number

31. For ewes that produced one or more deformed lambs please give us the number of lambings that:
were assisted by yourself or a farm hand

The value must be a number

32. For ewes that produced one or more deformed lambs please give us the number of lambings that:
were assisted by a vet

The value must be a number

33. For ewes that produced one or more deformed lambs please give us the number of lambings that:
required a caesarean

The value must be a number

Other Ruminants

34. Do you have any other ruminants on your property?

◯ Yes

◯ No

35. Which species do you have? Please write any that are not cattle or sheep in the "other" box

☐ cattle

☐ Other

36. Please write in the number of animals of each species

37. Have you had any aborted, stillborn or deformed young in any of these species?

◯ yes

◯ no

38. Please fill in the number (and species) of aborted still born or deformed young for the most recent birthing

39. Are there any other details on the 2023/24 lambing season you would like to share?

Virus Testing, Impact and Vaccination

40. Have you had prior testing done for Schmallenberg or Bluetongue?

- ○ yes
- ○ no

41. Please write in the year/s and virus you had testing done (if known)

42. What impact do you think Schmallenberg or Bluetongue have had on the welfare of your flock this lambing ?

	No impact	Some negative impact	Strong Negative Impact
Schmallenberg	○	○	○
Bluetongue	○	○	○

43. How much of an impact do you think Schmallenberg or Bluetongue will have on the financial performance of your sheep flock this year ?

	No impact	Some negative impact	Strong Negative Impact
Schmallenberg	○	○	○
Bluetongue	○	○	○

44. During lambing, how did the potential threat of Schmallenberg or Bluetongue impact you, your lambing staff, or your family in terms of emotional wellbeing?

	No impact	Some negative impact	Strong Negative Impact
Schmallenberg	○	○	○
Bluetongue	○	○	○

45. Has Schmallenberg or Bluetongue virus meant you are less likely to sheep farm next year?

- ◯ Yes Schmallenberg
- ◯ Yes Bluetongue
- ◯ Yes Both
- ◯ No
- ◯ Maybe

46. Have you ever vaccinated against either virus?

	Yes	No
Schmallenberg	◯	◯
Bluetongue (any strain)	◯	◯

47. Would you consider vaccinating your sheep for either virus if it was available now ? Please tick the highest price you would be prepared to pay.

	Would not vaccinate	Less than £1	Less than £2	Less than £3	Less than £4	More than £4
Schmallenberg	◯	◯	◯	◯	◯	◯
Bluetongue	◯	◯	◯	◯	◯	◯

48. Any other comments you would like to share ?

49. Please leave your email adress if you wish to receive a copy of the survey results and an update on Schmallenberg or Bluetongue impact and research

This content is neither created nor endorsed by Microsoft. The data you submit will be sent to the form owner.

Microsoft Forms
