# Supplementary material for: Survey to determine the farm‐level impact of Schmallenberg virus during the 2023–2024 UK lambing season
Source: Vet Rec. 2025 Aug 12;197(4):e5595. doi: 10.1002/vetr.5595 (PMC12355902; doi:10.1002/vetr.5595)
Supplement: Supplementary file 2 — Supporting Information [file VETR-197-e5595-s002.docx]

**Survey to determine the farm-level impact of Schmallenberg virus during the 2023–2024 United Kingdom lambing season: Supplementary information**


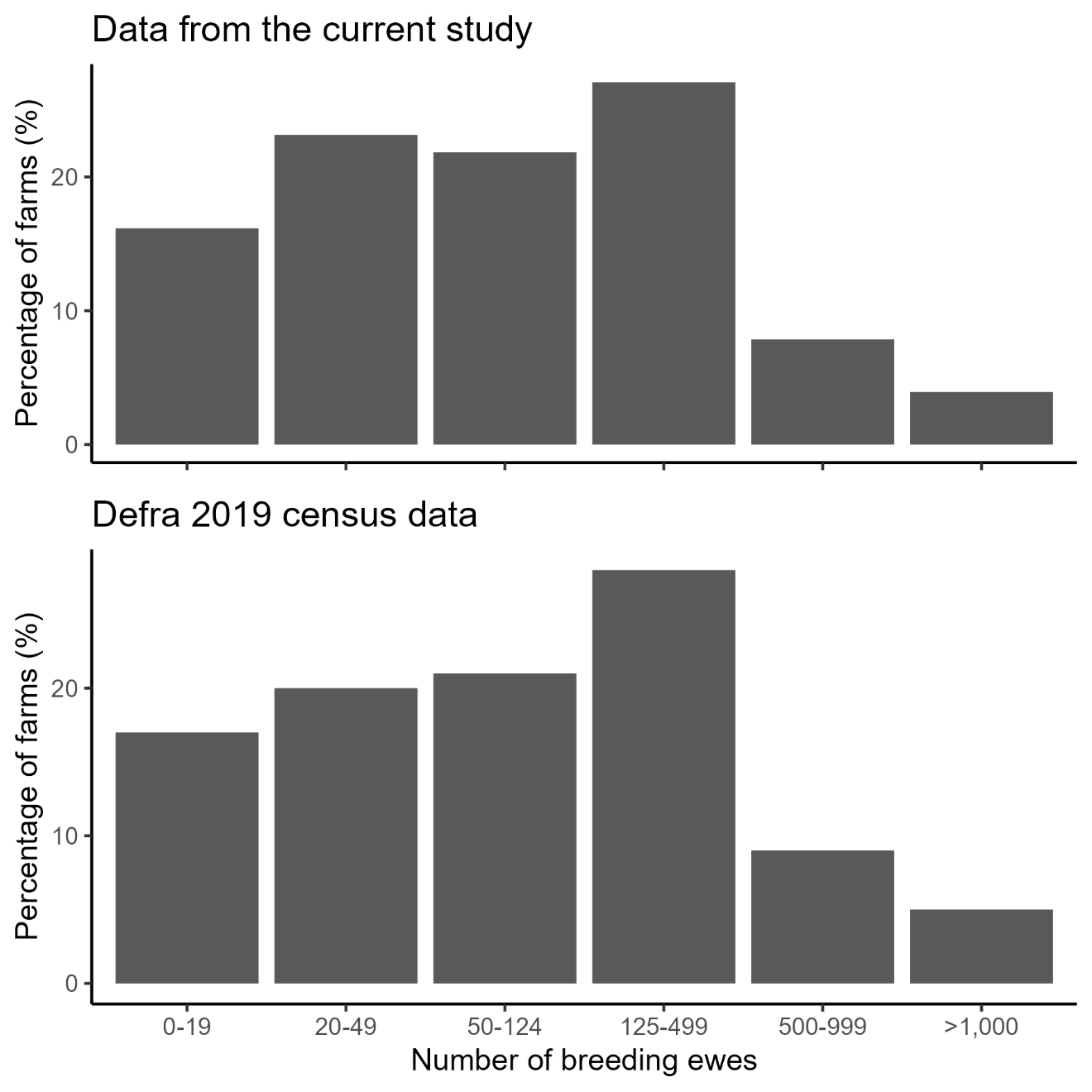


Figure S1 Distribution of flock size for respondents from the current study (upper panel) and sheep farms completing the 2019 Defra Agricultural Census (lower panel).

Table S1 Farm type and flock type by SBV category

|  | **SBV confirmed (n=44)** | | **SBV suspected (n=84)** | | **SBV not suspected (n=101)** | | **P values** |
| --- | --- | --- | --- | --- | --- | --- | --- |
|  | **n** | **%** | **n** | **%** | **n** | **%** |  |
| **Farm type*** |  |  |  |  |  |  | 0.074 |
| Responses (n) | 41 |  | 83 |  | 101 |  |  |
| Lowland (180) | 35 | 85.4% | 70 | 84.3% | 73 | 72.3% |  |
| Upland/hill (47) | 6 | 14.6% | 13 | 15.6% | 28 | 27.7% |  |
| **Flock type*** |  |  |  |  |  |  | 0.042 |
| Responses (n) | 44 |  | 84 |  | 101 |  |  |
| Crossbreeds/  Commercials (91) | 9 | 20.5% | 37 | 44.0% | 44 | 43.6% |  |
| Pedigree/pure bred (70) | 18 | 40.9% | 27 | 32.1% | 25 | 24.8% |  |
| Mix of both (70) | 17 | 38.6% | 20 | 23.8% | 32 | 31.7% |  |

*Farmers had to select one option to describe their flock. Percentages may not equal to 100 due to rounding. SBV, Schmallenberg virus.

Table S2 Farm breeding demographics by SBV category

|  | **SBV confirmed**  **n= 44** | **SBV suspected**  **n= 86** | **SBV not suspected n= 101** | **P values*** |
| --- | --- | --- | --- | --- |
| **Duration of mating season (days)** |  |  |  |  |
| Responses (n) | 44 | 79 | 98 |  |
| Earliest start date | 09 July 2023 | 01 July 2023 | 10 April 2023 |  |
| Latest end date | 19 February 2024 | 31 March 2024 | 25 May 2024 |  |
| Season duration |  |  |  | 0.013 |
| Median (days) | 59 | 58 | 48.5 |  |
| Min (days) | 1 | 14 | 2 |  |
| Max (days) | 192 | 196 | 196 |  |
| IQR (days) | 42.0-91.25 | 42.0-91.5 | 35.0-65.75 |  |
| **Duration of lambing** |  |  |  |  |
| Responses (n) | 41 | 81 | 98 |  |
| Earliest start date | 04 December 2023 | 12 December 2023 | 04 September 2023 |  |
| Latest end date | 07 July 2024 | 30 June 2024 | 01 July 2024 |  |
| Season duration |  |  |  | 0.002 |
| Median (days) | 35 | 50 | 35 |  |
| Min (days) | 4 | 3 | 10 |  |
| Max (days) | 188 | 178 | 100 |  |
| IQR (days) | 28.0-72.0 | 33.0-64.0 | 28.0-46.5 |  |
| **Tupped ewes that were barren (%)** |  |  |  | 0.055 |
| Responses (n) | 39 | 64 | 75 |  |
| Median | 7.3% | 4.8 | 3.5 |  |
| Min | 0 | 0 | 0 |  |
| Max | 33.3% | 34.0% | 33.3 |  |
| IQR | 2.3-10.9 | 2.4-8.4 | 1.6-5.7 |  |
| **Lambing percentage (%)** |  |  |  | 0.03 |
| Responses (n) | 34 | 58 | 61 |  |
| Median | 146.6% | 153.8 | 168.6 |  |
| Min | 16.7 | 5.8 | 5.1 |  |
| Max | 220.7 | 228.6 | 221.4 |  |
| IQR | 127.1-163.9 | 119.0-170.2 | 140.0-183.1 |  |
| **Scanning percentage (%)** |  |  |  | 0.21 |
| Responses (n) | 37 | 62 | 74 |  |
| Median | 165.0 | 170.0 | 168.5 |  |
| Min | 80 | 123 | 100 |  |
| Max | 210 | 250 | 220 |  |
| IQR | 134.0-180.0 | 157.0-185.0 | 152.25-189.5 |  |

*Kruskal Wallis tests were conducted except where data were normally distributed in which case ANOVA was conducted. Dunn test was performed for all significant Kruskal Wallis tests to determine pairwise differences between groups. SBV, Schmallenberg virus.

Table S3 Lamb mortality and lambing mortality by SBV category

|  | **SBV confirmed n= 44** | **SBV suspected n= 84** | **SBV not suspected n= 101** | **P values*** |
| --- | --- | --- | --- | --- |
| **Lamb mortality (%) (per lambs born)** |  |  |  | <0.001 |
| Responses (n) | 40 | 77 | 82 |  |
| Median | 19.6 | 15.4 | 7.6 |  |
| Min | 1.4 | 0 | 0 |  |
| Max | 100 | 100 | 87.5 |  |
| IQR | 10.1-26.0 | 7.1-31.8 | 4.2-13.9 |  |
| **Lambing mortality (%) (per pregnant ewes)** |  |  |  | <0.001 |
| Responses (n) | 38 | 60 | 72 |  |
| Median | 27.2 | 15.9 | 10.4 |  |
| Min | 1.9 | 1.4 | 0 |  |
| Max | 62.1 | 100 | 54.9 |  |
| IQR | 14.6-33.7 | 11.4-29.4 | 5.2-16.3 |  |

*Kruskal Wallis tests were conducted except where data were normally distributed in which case ANOVA was conducted. SBV, Schmallenberg virus. Dunn test was performed for all significant Kruskal Wallis tests to determine pairwise differences between groups. SBV, Schmallenberg virus.

Table S4 Ewe mortality and assisted births by SBV category

|  | **SBV confirmed n= 44** | | **SBV suspected n= 86** | | **SBV not suspected n= 101** | | **P values** |
| --- | --- | --- | --- | --- | --- | --- | --- |
|  | **n** | **%** | **n** | **%** | **n** | **%** |  |
| **Breeding ewes that died during the lambing period** |  |  |  |  |  |  | 0.5 |
| Responses (n) | 42 |  | 84 |  | 100 |  |  |
| 0 | 22 | 52.4% | 36 | 42.9% | 42 | 42% |  |
| ≥1 | 20 | 47.6% | 48 | 57.1% | 58 | 58% |  |
| **Ewes that died giving birth to a deformed lamb** |  |  |  |  |  |  | 0.005 |
| Responses (n) | 43 |  | 83 |  | 98 |  |  |
| 0 | 29 | 67.4% | 61 | 73.5% | 87 | 88.8% |  |
| ≥1 | 14 | 32.6% | 22 | 26.5% | 11 | 11.2% |  |
| **Ewes that gave birth to deformed lambs alone** |  |  |  |  |  |  | 0.12 |
| Responses (n) | 41 |  | 81 |  |  |  |  |
| 0 | 18 | 43.9% | 48 | 59.3% | NA | NA |  |
| ≥1 | 23 | 56.1% | 33 | 40.7% | NA | NA |  |
| **Ewes assisted by farmer because of a deformed lamb** |  |  |  |  |  |  | 0.07 |
| Responses (n) | 43 |  | 81 |  |  |  |  |
| 0 | 3 | 7.0% | 16 | 19.8% | NA | NA |  |
| ≥1 | 40 | 93.0% | 65 | 80.2% | NA | NA |  |
| **Ewes assisted by vet because of a deformed lamb** |  |  |  |  |  |  | 0.0003 |
| Responses (n) | 43 |  | 82 |  |  |  |  |
| 0 | 20 | 46.5% | 65 | 79.3% | NA | NA |  |
| ≥1 | 23 | 53.5% | 17 | 20.7% | NA | NA |  |
| **Caesarean sections because of deformed lamb** |  |  |  |  |  |  | 0.006 |
| Responses (n) | 43 |  | 82 |  |  |  |  |
| 0 | 29 | 67.4% | 73 | 89.0% | NA | NA |  |
| ≥1 | 14 | 32.6% | 9 | 11.0% | NA | NA |  |

Percentages may not add to 100 due to rounding. SBV, Schmallenberg virus.

Table S5 Perceived impact of SBV on the flocks’ welfare, the financial performance of flocks, the farmers’ emotional wellbeing and whether the respondent intends to give up sheep farming due to the impact of SBV this year by SBV category

|  | **SBV confirmed n= 44** | | **SBV suspected n= 86** | | **SBV not suspected n= 101** | | **P values** |
| --- | --- | --- | --- | --- | --- | --- | --- |
|  | **n** | **%** | **n** | **%** | **n** | **%** |  |
| Responses (n) | 43 |  | 84 |  | 99 |  |  |
| **Impact of SBV on sheep flocks’ welfare** |  |  |  |  |  |  | <0.001 |
| No impact | 0 | 0.0% | 6 | 7.1% | 91 | 91.9% |  |
| Strong positive impact | 0 | 0.0% | 0 | 0.0% | 0 | 0.0% |  |
| Some positive impact | 0 | 0.0% | 0 | 0.0% | 0 | 0.0% |  |
| Some negative impact | 16 | 37.2% | 46 | 54.8% | 4 | 4.0% |  |
| Strong negative impact | 27 | 62.8% | 32 | 38.1% | 4 | 4.0% |  |
| **Impact of SBV on sheep flocks’ financial performance** |  |  |  |  |  |  | <0.001 |
| No impact | 0 | 0.0% | 5 | 6.0% | 84 | 84.8% |  |
| Strong positive impact | 0 | 0.0% | 0 | 0.0% | 0 | 0.0% |  |
| Some positive impact | 0 | 0.0% | 0 | 0.0% | 0 | 0.0% |  |
| Some negative impact | 13 | 30.2% | 45 | 54.2% | 11 | 11.1% |  |
| Strong negative impact | 30 | 69.8% | 33 | 39.8% | 4 | 4.0% |  |
| **Impact of SBV on farmers’ emotional wellbeing** |  |  |  |  |  |  | <0.001 |
| No impact | 0 | 0.0% | 9 | 10.7% | 56 | 56.6% |  |
| Strong positive impact | 0 | 0.0% | 0 | 0.0% | 0 | 0.0% |  |
| Some positive impact | 0 | 0.0% | 0 | 0.0% | 0 | 0.0% |  |
| Some negative impact | 12 | 27.9% | 39 | 46.4% | 34 | 34.3% |  |
| Strong negative impact | 31 | 72.1% | 36 | 42.9% | 9 | 9.1% |  |
| **Less likely to sheep farm next year because of SBV/BTV** |  |  |  |  |  |  | 0.08 |
| Maybe | 8 | 18.6% | 13 | 15.5% | 8 | 8.1% |  |
| No | 31 | 72.1% | 64 | 76.2% | 88 | 88.9% |  |
| Yes both | 0 | 0.0% | 1 | 1.2% | 2 | 2.0% |  |
| Yes SBV | 4 | 9.3% | 6 | 7.1% | 1 | 1.0% |  |

Percentages may not add to 100 due to rounding. SBV, Schmallenberg virus.

Table S6 Respondents’ willingness to vaccinate against SBV at different prices for different SBV categories

|  | **SBV confirmed n= 44** | | **SBV suspected n= 86** | | **SBV not suspected n= 101** | | **P values** |
| --- | --- | --- | --- | --- | --- | --- | --- |
|  | **n** | **%** | **n** | **%** | **n** | **%** |  |
| **Would you consider vaccinating your sheep against Schmallenberg virus if it was available now?** |  |  |  |  |  |  | <0.001 |
| Responses (n) | 43 |  | 84 |  | 99 |  |  |
| Would not vaccinate | 1 | 2.3% | 4 | 4.8% | 16 | 16.2% |  |
| Yes, if it costs less than £1 | 6 | 14.0% | 16 | 19.0% | 25 | 25.3% |  |
| Yes, if it costs less than £2 | 12 | 27.9% | 30 | 35.7% | 33 | 33.3% |  |
| Yes, if it costs less than £3 | 10 | 23.3% | 9 | 10.7% | 13 | 13.1% |  |
| Yes, if it costs less than £4 | 6 | 14.0% | 9 | 10.7% | 5 | 5.1% |  |
| Yes, would pay more than £4 | 8 | 18.6% | 16 | 19.0% | 7 | 7.1% |  |

Percentages may not add to 100 due to rounding. SBV, Schmallenberg virus

**Regression models to check for confounding by mating start date**

Multivariable linear regression models were built using the lm() function in R and included SBV category and month that mating started as categorical explanatory variables. For SBV category, SBV not suspected was used as the reference category. For month mating started, the months March-July were grouped as one category as there were very few flocks for each of these months. This resulted in 5 categories: March-July (n=14), August (n=66), September (n=51), October (n=66) and November (n=27). October was used as the reference category.

Outcome variables analysed using these models were: empty rate, scanning percentage, lambing percentage, lamb mortality and lambing mortality. Non-normal outcome variables were transformed for regression analysis (normality visually assessed using histograms). Model fit was checked using residual versus fitted and Q-Q residuals plots.

ANOVA tables of the resultant models were called using the anova() function in R and used to determine the overall significance level of the association between SBV category and the outcome variable of interest. Results for each outcome variable are shown in Table S7.

Table S7 ANOVA tables for associations between outcome variables and SBV category whilst accounting for confounding by mating start date

| **Outcome variable** | **Degrees of freedom** | **F value** | **P value** |
| --- | --- | --- | --- |
| **Empty rate** |  |  |  |
| SBV category | 2 | 2.60 | 0.08 |
| Month mating started | 4 | 3.66 | <0.01 |
| Residuals | 168 |  |  |
| **Scanning percentage** |  |  |  |
| SBV category | 2 | 1.73 | 0.18 |
| Month mating started | 4 | 1.81 | 0.13 |
| Residuals | 160 |  |  |
| **Lambing percentage** |  |  |  |
| SBV category | 2 | 3.02 | 0.05 |
| Month mating started | 4 | 0.70 | 0.59 |
| Residuals | 143 |  |  |
| **Lamb mortality** |  |  |  |
| SBV category | 2 | 11.1 | <0.001 |
| Month mating started | 4 | 1.39 | 0.23 |
| Residuals | 188 |  |  |
| **Lambing mortality** |  |  |  |
| SBV category | 2 | 17.4 | <0.001 |
| Month mating started | 4 | 2.93 | 0.02 |
| Residuals | 160 |  |  |
